# Non-albicans Candida Infection as a Rare Cause of Emphysematous Pyelonephritis in an Uncontrolled Diabetic Patient: A Case Report

**DOI:** 10.7759/cureus.57036

**Published:** 2024-03-27

**Authors:** Pranav Chaudhari, Rucha Sawant, Meghna Bordoloi, Sunil Kumar, Sourya Acharya

**Affiliations:** 1 Medicine, Jawaharlal Nehru Medical College, Wardha, IND

**Keywords:** non-albicans candida, complicated pyelonephritis, severe sepsis, insulin use, diabetes mellitus type 2, emphysematous infection

## Abstract

The uncommon but dangerous condition known as emphysematous pyelonephritis (EPN) usually affects people with diabetes. This potentially fatal illness is characterized by gas-forming necrosis of the kidneys and surrounding tissues, typically brought on by urinary tract bacteria. Fungal EPN, less prevalent than bacterial EPN, has been reported in a few isolated cases. Cultures of the urine or blood often detect the infection. With an 18% fatality rate, EPN is still a serious illness despite advancements in therapy. High suspicion for EPN is critical in diabetic patients experiencing pyelonephritis. Interestingly, women with uncontrolled diabetes seem to be more susceptible. While *Escherichia coli* is the usual culprit, rare cases involve *Candida *species. This case report describes a pathogen that is rarely encountered and causes EPN. A diabetic woman in her sixties without prior hospitalizations presented with a sudden fever and excruciating abdominal pain. The patient also complained of abdominal distension with reduced urine output and breathlessness at rest. Investigations revealed left-sided EPN that was “WAN Type 1.” We treated the patient according to culture sensitivity with systemic antifungals, percutaneous nephrostomy (PCN), and ureteral stenting (double J stent or DJ stent). Following successful treatment, the patient recovered and was discharged. This case highlights the importance of considering uncommon causes, even in seemingly typical presentations of EPN. Our case is unique as the patient had an infection with non-albicans Candida with a complication of anuric acute kidney injury and uncontrolled diabetes mellitus.

## Introduction

Emphysematous pyelonephritis (EPN) is a rare but serious necrotizing kidney infection characterized by gas-forming tissue destruction [1]. Typically caused by common urinary tract bacteria, it carries a high risk of death for patients with certain risk factors [1,2]. Investigating less common causes is crucial if patients fail to respond to standard antibiotic treatment or worsen. Several bacteria, including *Escherichia coli*, *Klebsiella pneumoniae*, *Proteus mirabilis*, and occasionally fungi like *Candida albicans*, can trigger EPN [3]. In a favorable environment like uncontrolled diabetes, these organisms ferment glucose and lactose, creating gas and causing tissue death in the kidney's surrounding fat [4].

Notably, nearly 90% of EPN cases occur in people with poorly managed diabetes [5,6], especially women (3:1), often affecting the left kidney [7]. This is because the implicated organisms like *E. coli* thrive in hyperglycemia and glycosuria. The mainstay for extensive disease treatment is nephrectomy [7]. This medical condition warrants additional focus within intensive care literature due to its dire prognosis and the potential life-saving advantages of prompt diagnosis and treatment. It is prudent to consider this condition in any diabetic patient showing signs of septicemia or pyelonephritis.

A 64-year-old woman having type 2 diabetes mellitus presented with EPN, as confirmed with a CT scan and lab tests. This case is noteworthy for two reasons, i.e., it represents, to the best of our knowledge, one of the very few documented instances of non-albicans Candida causing EPN in a diabetic, and the patient was managed without nephrectomy and only by percutaneous nephrostomy (PCN) and ureteral stenting.

## Case presentation

A female in her sixties, with a known case of systemic hypertension and diabetes mellites type 2 for three years on angiotensin receptor blocker (telmisartan 40 mg once a day) and biguanide (metformin 500 mg twice a day) presented to the casualty with complaints of excruciating abdominal pain with acute onset high-grade fever associated with chills, abdominal distension, and difficulty in breathing. The patient also complained of oliguria in the past three days and was initially catheterized with a urinary Foley catheter. On initial examination, the patient was febrile with 101°F, 110/min heart rate, a respiratory rate of 28 breaths/min, and a blood pressure of 100/70 mmHg in the right arm in the supine position. Signs of congestive heart failure were absent. A systemic examination revealed tenderness in the left lumbar area and the left renal angle, and a palpable mass was felt in the left lumbar region.

Laboratory (Lab) parameters (Table 1) suggested microcytic hypochromic anemia and a hemoglobin (Hb) of 10.1 g/dL, a platelet count of 94000/mm^3^, and a total leukocyte count (TLC) of 18,000/mm^3^ with 80% of neutrophils. Urine microscopy revealed 40-50 pus cells per high power field, positive for urinary sugar and albumin +, and negative for ketone bodies. A pH of 7.3, sodium of 128 mmol/L, potassium of 2.5 mmol/dL, calcium of 7.1 mg/dL, partial pressure of carbon dioxide (PCO2) of 38 mmHg, partial pressure of oxygen (PO2) of 110 mmHg, an oxygen saturation of the arterial blood (SaO2) of 94% on room air, and bicarbonate (HCO3) of 15 mmol/L was seen on arterial blood gas analysis. Random blood sugar readings were persistently present in the range of 450-550 mg/dL despite treatment with regular subcutaneous insulin. Glycosylated hemoglobin (HbA1c) was 12.2%. The patient has a BMI of 29.

**Table 1:** Laboratory investigations. Sr, serum; RBS, random blood sugar; HbA1c, glycosylated hemoglobin; dHDL, direct high-density lipoprotein

PARAMETERS	INITIAL VALUES	POST-TREATMENT VALUES	NORMAL RANGE
Hemoglobin (gm/dL)	10.1	13.1	11-15
Total white blood cell count (per mm^3^)	18000	8700	4000-10000
Total red blood cell count (million per mm^3^)	3.88	4.01	4.2-5.4
Platelets (per mm^3^)	94000	233000	140000-440000
Hematocrit (%)	35.5	38	36-46
Urea (mg/dL)	92	48	10-45
Creatinine (mg/dL)	3.2	1.1	0.2-1.2
Sodium (meq/L)	130	138	135-148
Potassium (meq/L)	2.7	4	3.5-5.3
Magnesium (mg/dL)	1.5	1.9	1.6-2.3
Calcium (mg/dL)	7.7	9.6	8.4-10.2
Sr. ionized calcium (mEq/L)	4.32	4.56	4.4-5.2
Phosphorus (mg/dL)	3.9	4.8	2.5-4.5
Uric acid (mg/dL)	8.6	3	2.5-6.5
RBS (mg/dL)	526	160	<140
Serum glutamyl-oxaloacetate transaminase (U/L)	64	60	11.8-64.8
Serum glutamyl-pyruvate transaminase (U/L)	26	46	8.5-49.5
Albumin (mg/dL)	2.7	3.8	3.2-5.1
Total bilirubin (mg/dL)	0.6	0.6	0-0.8
International normalized ratio (INR)	1.25	1.2	1.3-1.5
HbA1c (%)	12.2	-	<6
Vitamin D (ng/mL)	18.8	26	>30
Total cholesterol (mg/dL)	96	96	Desirable <200; borderline 200-239; high >240
Triglycerides (mg/dL)	462	250	Normal <150; borderline 150-199; high >200
dHDL (mg/dL)	24	36	Low <40; high >60

**Figure 1:**
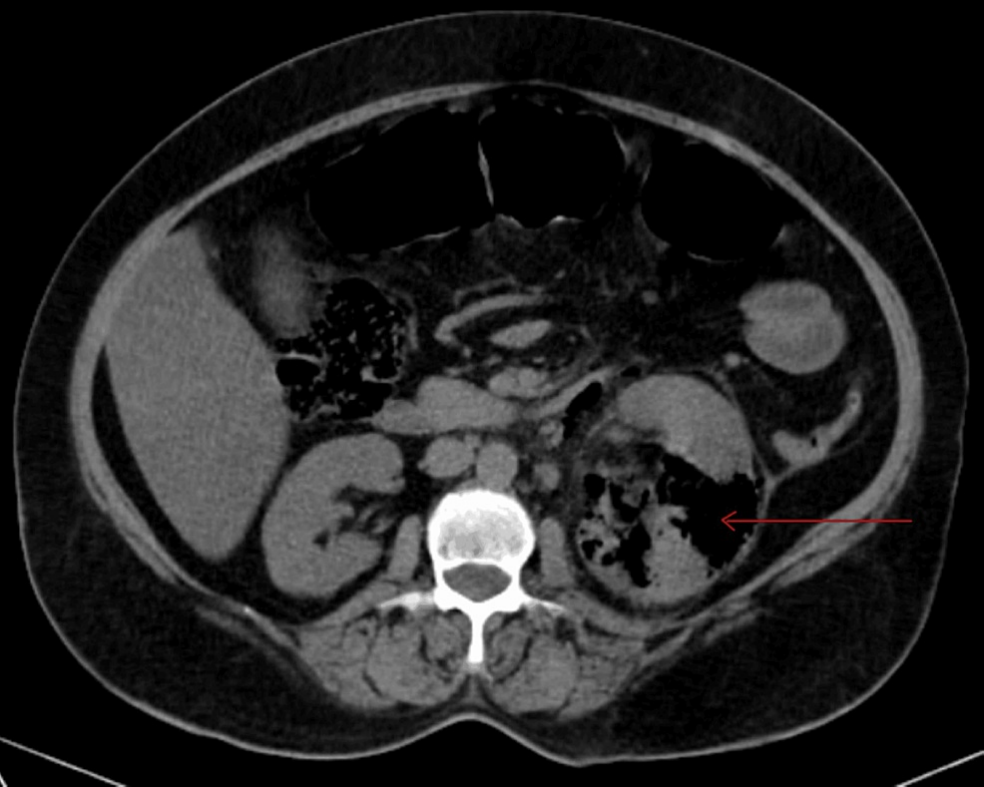
Non-contrast computed tomography showing the left kidney with loss of normal architecture with air foci and perinephric fat stranding.

Ultrasonography (USG) revealed an enlarged left kidney (12.8 × 6.1 cm) with raised echotexture and multiple air foci within the renal parenchyma and pelvicalyceal system. The right kidney was normal (10 × 5 cm). Hepatomegaly with a liver span of 17 cm with raised echotexture and grade I hepatomegaly was present. An abdominal non-contrast computed tomography (NCCT) scan suggested left renal parenchyma with extensive air pockets and pelvicalyceal system with perinephric fat stranding and enlarged lymph nodes adjacent to it (Figure 1).

**Figure 2:**
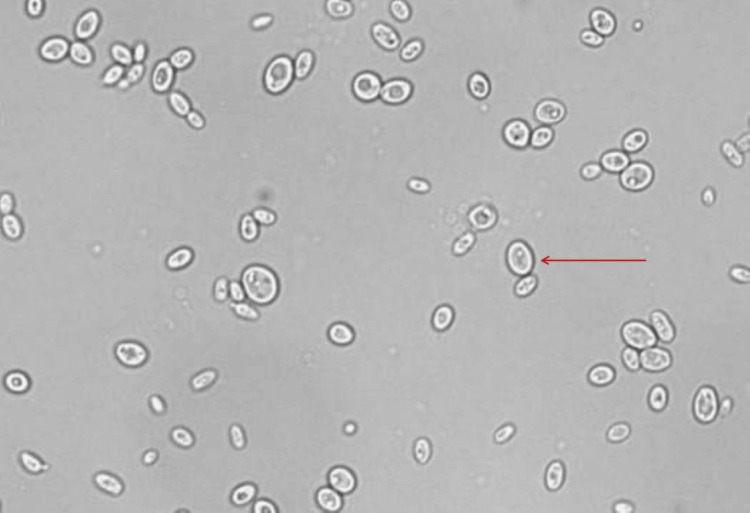
Urine culture microscopy image showing non-albicans Candida.

**Figure 3:**
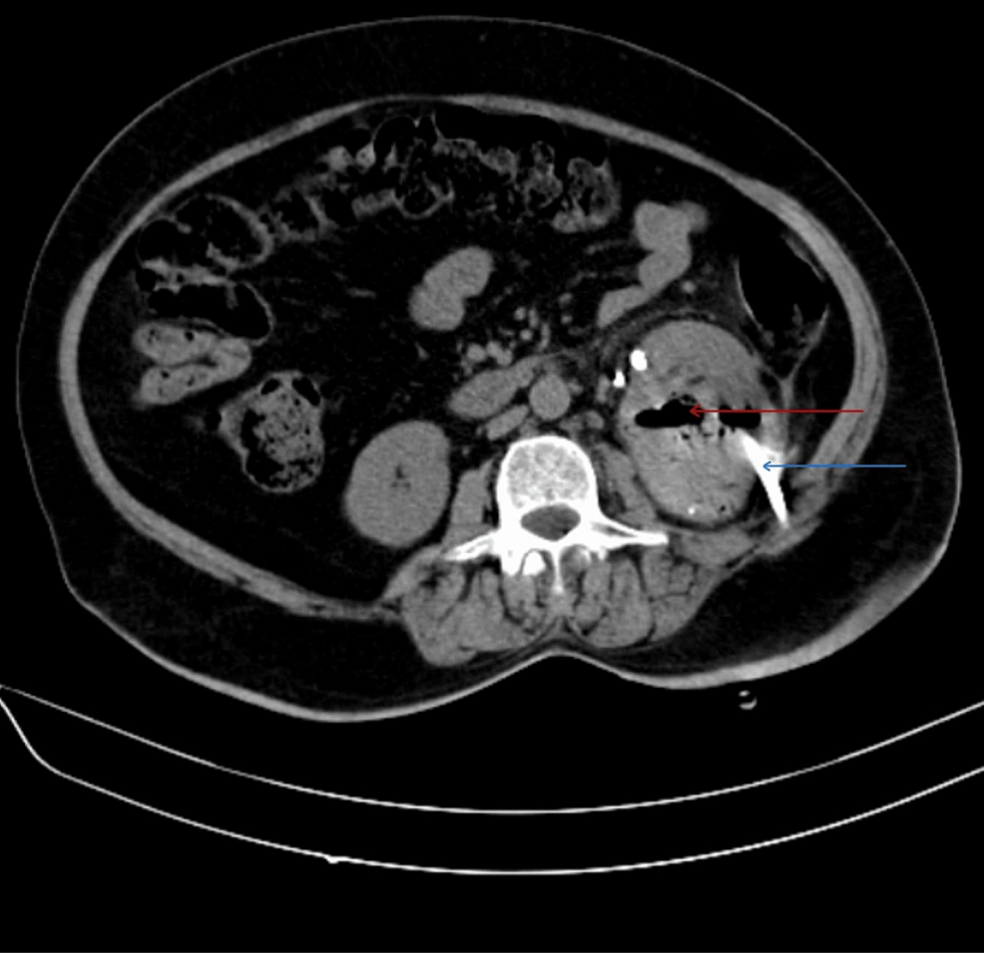
Non-contrast computed tomography of the abdomen showing the left kidney appearing enlarged with air foci and fluid attenuation collection within it (red arrow) with percutaneous nephrostomy catheter seen in-situ (blue arrow).

**Figure 4:**
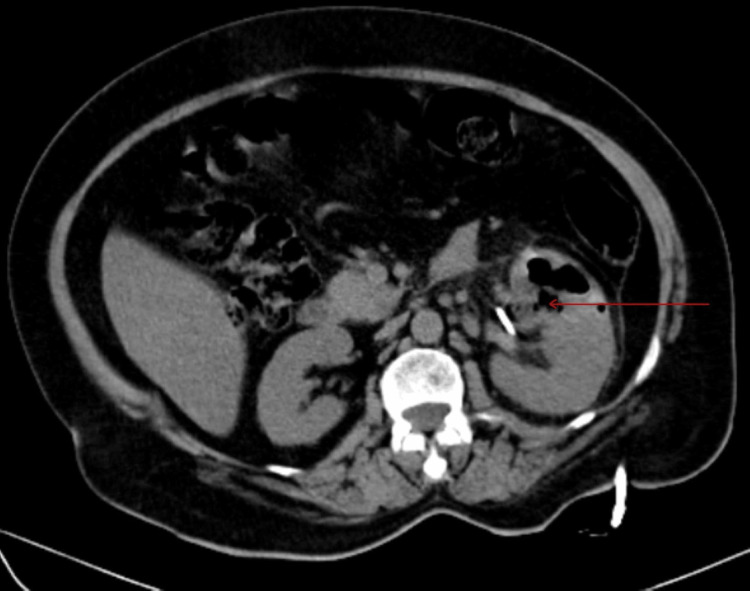
Non-contrast computed tomography of the abdomen showing reduction in air pockets within the left renal parenchyma after six days of initial scan.

**Figure 5:**
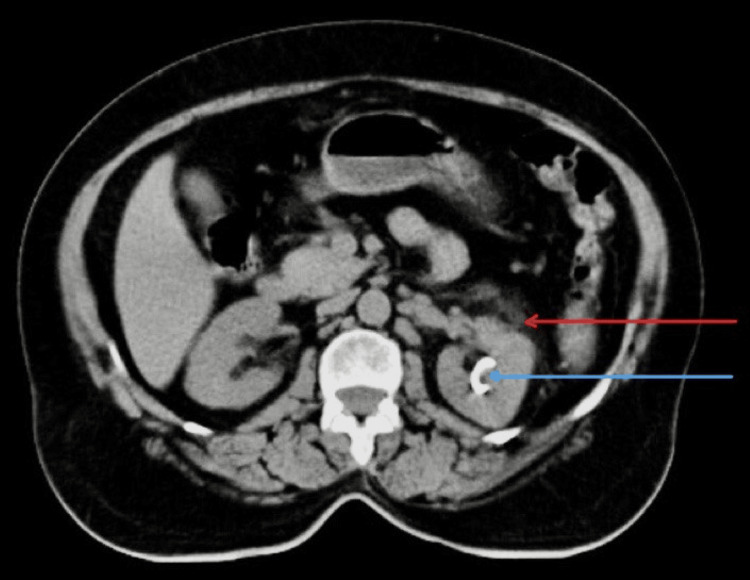
Non-contrast computed tomography of the abdomen (four months post initial scan) showing the left renal parenchyma with urethral stent in-situ (blue arrow) and left-sided perinephric fat stranding (red arrow) with no intrarenal or perirenal air foci noted on the scan.

Injectable antibiotics (metronidazole, piperacillin-tazobactam, linezolid) were started, prior to which cultures of urine and blood were sent. The culture came out positive for non-albicans Candida, and repeat subsequent cultures tested positive for the same (Figure 2). On sensitivity testing, the fungus was sensitive to fluconazole. Hence, intravenous (IV) fluconazole 200 mg twice a day was given. The patient was treated with regular subcutaneous insulin, which was a daily requirement in the range of 90-110 units. Symptomatic treatment was given with daily IV fluids, strict intake-output charting, and electrolyte correction. Initially, the patient was afebrile for two days after starting treatment; creatinine was reduced to 2.2 mg/dL, and the liver function test improved with 3.8 g/dL albumin and total protein of 7.2 g/dL. However, the patient continued to have abdominal pain and other symptoms, and creatinine was static at 2.2 mg/dL. Hence, the patient was advised of ureteral stenting and PCN, which was done seven days after admission. The patient improved drastically with TLC of 9000/mm^3^ and kidney function improvement. Repeat CT abdomen was done a week after PCN and ureteral stenting and suggested left kidney was enlarged and measuring 12.1 x 5.5 with few variable-sized air pockets in the left renal parenchyma and pelvicalyceal system and suggested reduction of air pockets in left parenchyma as compared to previous scan (Figure 3 and Figure 4). Lab investigations at discharge suggested Hb of 13.1 g/dL, TLC, which improved to 8700/mm^3^ (polymorphocytes, 70%; lymphocytes, 18%) (Table 1). Repeat blood and urine cultures were sterile. After being hospitalized for five weeks, the patient's symptoms significantly subsided. The patient's clinical status was deemed adequate at discharge. Subsequent follow-up after four months of discharge, CT abdomen (Figure 5) showed left-sided perinephric fat stranding with a small pocket of fluid collection with no perirenal or intrarenal air foci in the scan.

## Discussion

EPN is a highly destructive renal infection characterized by tissue death and gas formation [8]. Its initial description dates back to 1898, with the term "emphysematous pyelonephritis" coined in 1962 [9]. High mortality risk has been documented up to 40%-50% [10]. The term "emphysematous pyelonephritis" was initially used in 1962 by Schultz and Klorfein to delineate an acute infectious disease that caused gas production in the renal parenchyma [11]. Numerous reasons, including elevated tissue sugar levels, compromised immune systems, tissue ischemia, and fermentation of mixed acids by gas-producing organisms, have been proposed as causes of gas generation. Predominantly, the risk factor for end-stage renal disease (EPN) is uncontrolled diabetes [12]. Additional risk factors include urinary tract blockage, polycystic kidneys, end-stage renal disease, and immunosuppression [1].

EPN is an uncommon infection of bacteria known to produce gas and typically colonize the urinary tract and include *Proteus mirabilis*, *E. coli*, *Klebsiella pneumoniae*, and *Pseudomonas aeruginosa* [13,14]. Additionally, certain *Candida *species, such as* Candida guilliermondii*, *Candida parapsilosis*, and *Candida albicans*, have demonstrated the ability to produce gas in laboratory settings [15]. Detecting fungi in the laboratory often involves conducting a sugar fermentation test, where a positive result indicates the presence of acid and gas in the test tube [16]. Although rare, a limited number of case reports attribute fungi as an etiological factor [15,17-19].

The clinical manifestations of pyelitis and EPN closely resemble those observed in severe, acute pyelonephritis. Typically, patients present with symptoms such as fever with chills, flank or abdomen discomfort, and vomiting. These symptoms can manifest suddenly or develop gradually over a period of two to three weeks.

Acute anuric kidney damage is a rare side effect of EPN, affecting individuals with unilateral illness affecting a single kidney or bilateral infection [20].

The most effective tool for diagnosing EPN is a CT scan [13,14]. Two kinds of EPN were defined by Wan et al. based on CT results [21]. In this study, type I patients, having 69% mortality, showed parenchymal damage with mottled gas without any fluid accumulation, while type II patients, having 18% mortality, had renal or perirenal fluid accumulation with loculated gas or gas within the collecting system. In our case, the patient's traits aligned with type 1.

X-ray erect abdomen has a sensitivity of 50-85% in the detection of air associated with emphysematous UTI [22]. Similarly, a comprehensive study found that renal ultrasonography had a 68% accuracy rate in identifying EPN [22].

Emphysematous pyelitis or pyelonephritis is difficult to clinically differentiate from acute severe pyelonephritis, renal abscess, or acute papillary necrosis. Unlike EPN, emphysematous cystitis sometimes has an unusual presentation where the extent of inflammation does not correlate with a spectrum of clinical symptoms [23], although radiologically, these diseases can be distinctly differentiated. Renal abscess with air, gas-producing organisms causing psoas abscess, cutaneo-renal or entero-renal fistula, air reflux from the bladder, abdominal retroperitoneal perforation, and recent intervention such as nephrostomy insertion are among the conditions that are included in the possible causes for a gathering of air within or near the kidney parenchyma. To differentiate between these conditions, CT findings can be useful.

Extensive EPN having certain complications such as acute kidney injury, thrombocytopenia, low-sodium, confusion, and septic shock has been associated with high mortality. Multiple prognostic classifications have been proposed for EPN. The most commonly used system uses CT scan imaging to categorize EPN into four classes, with higher classes associated with poorer outcomes [1]. A categorization approach included the extent of the disease with possible risk factors for unfavorable consequences [24]. According to this, one point each was allotted for the following: age >50 years; total leukocyte count ≥12,000 or ≤4000; BMI ≥30 or ≤18; platelets ≤100,000/μL; serum creatinine ≥3; albumin ≤2.5 g/dL; class 2 or 3 disease; sodium ≤130; ≥2 comorbidities; and multidrug-resistant organism.

Based on the score, patients can be further divided into three groups, i.e., good, intermediate, and poor categories, with scores of 0-4, 5-7, and >7, respectively. According to this study, patients required emergency nephrectomy after antibiotics and percutaneous drainage with a mortality of 2% in the good category, 19% in the intermediate category, and 100% in the poor category [24]. According to this, our patient had a score of 5.

## Conclusions

A severe complication found generally in diabetics and requiring prompt diagnosis and treatment for good prognosis is EPN. Most commonly, uropathogens are implicated, although other organisms, such as fungi, can be causative agents either alone or in coinfection with other organisms in decompensated diabetes mellitus. Intervention depends on the extent of the disease, clinical status, and presence of complications. Early detection by CT scan is crucial, along with medical management with antibiotics, percutaneous drainage, and nephrectomy, which form the mainstay of treatment as indicated by disease classification and risk factors. Culture of both blood and urine, along with sensitivity, should be obtained for all cases, and antibiotics should be started empirically, as in our case, followed by culture-isolated organism sensitivity-specific antibiotics. The primary intention in all cases should be to reduce the high morbidity and mortality seen in this disease.
